# Fabrication and Properties of InGaZnO Thin-Film Transistors Based on a Sol–Gel Method with Different Electrode Patterns

**DOI:** 10.3390/mi13122207

**Published:** 2022-12-13

**Authors:** Xingzhen Yan, Bo Li, Kaian Song, Yiqiang Zhang, Yanjie Wang, Fan Yang, Chao Wang, Yaodan Chi, Xiaotian Yang

**Affiliations:** Key Laboratory of Architectural Cold Climate Energy Management, Ministry of Education, School of Electronics and Computer, Jilin Jianzhu University, 5088 Xincheng Street, Changchun 130118, China

**Keywords:** thin-film transistor, metal oxide, InGaZnO channel, circular electrodes, solution-processed

## Abstract

The preparation of thin-film transistors (TFTs) with InGaZnO (IGZO) channels using sol–gel technology has the advantages of simplicity in terms of process and weak substrate selectivity. We prepared a series of TFT devices with a top contact and bottom gate structure, in which the top contact was divided into rectangular and circular structures of drain/source electrodes. The field-effect performance of TFT devices with circular pattern drain/source electrodes was better than that with a traditional rectangular structure on both substrates. The uniform distribution of the potential in the circular electrode structure was more conducive to the regulation of carriers under the same channel length at different applied voltages. In addition, with the development of transparent substrate devices, we also constructed a hafnium oxide (HfO_2_) insulation layer and an IGZO active layer on an indium tin oxide conductive substrate, and explored the effect of circular drain/source electrodes on field-effect properties of the semitransparent TFT device. The IGZO deposited on the HfO_2_ dielectric layer by spin-coating can effectively reduce the surface roughness of the HfO_2_ layer and optimize the scattering of carriers at the interface in TFT devices.

## 1. Introduction

With the gradual upgrading of electronic devices, the performance of display components should also meet the demand of the industry, among which thin-film transistors (TFTs) are an important part of screen display with broad application prospects [1,2,3]. The low-cost, highly reliable, and low-power-consumption TFTs in functional integrated circuits have received increasing attention, and the material selection and structure design of each functional layer in devices are further improved to enhance field-effect properties [4,5,6,7,8]. Metal oxides [9,10,11,12,13,14] and organic semiconductors [15,16,17] are two promising classes of TFT channel materials that have made impressive progress in TFT devices compared to traditional silicon. Organic active-layer TFTs offer potential use in large-area electronic display devices [18,19]. However, inorganic channel layers can maintain good thermal and electrical stability and avoid affecting the device performance caused by the degradation of material itself during long operation cycles [20,21]. Moreover, metal oxide TFTs are developed mainly for display driven applications, due to their advantages of stable material properties, controllable element doping, and simple preparation process [22].

Among metal oxide semiconductors, the amorphous InGaZnO (IGZO) system is especially promising for use as a high-performance TFT active material. Because the indium as a doping cation affects the electronic configuration in channel layers, and the stability of the gallium–oxygen bond suppresses the generation of oxygen vacancies, thus decreasing the free electron concentration [9,23,24,25]. The traditional preparation method of the metal oxide active layer relies on vacuum deposition technology [26]. Although sputtering deposition is beneficial to the quality of films, it cannot meet the demand of the current device fabrication process simplification. Sol–gel technology has the advantages of low cost, diversified choices of doping materials, and controllable component ratio in a simple preparation process [27]. Moreover, the metal oxide films prepared by spin-coating method have a wide selection of substrates [28]. The premise is to improve the wetting of the precursor solution onto the target substrates [29].

Different types of TFTs were formed by spin-coating IGZO on a traditional Si/SiO_2_ substrate and a transparent substrate composed of an indium tin oxide (ITO) film and a hafnium dioxide (HfO_2_) as the bottom gate and insulation layer. Transparent metal oxide TFTs have been widely studied in wearable electronic devices and smart displays [30]. Moreover, with the improvement in the performance of TFT devices, the circular electrode structure should be explored to break the traditional rectangular symmetrical structure in TFTs. The circular electrode structure can solve the difference of electric field distribution between electrode centers and electrode edges compared with a traditional rectangular symmetrical structure. In this study, a spin-coating process that can prepare InGaZnO channel and also reduce the roughness of the interface between the active and the dielectric layer for TFTs was reported. A TFT structure with a bottom gate consisting of Si wafers or ITO conductive glass and a top contact consisting of rectangular symmetrical and circular drain/source electrodes was constructed to analyze the difference in electrical properties of TFT devices. In addition, the introduction of circular drain/source electrodes to optimize the uniformity of electric field distribution and the field-effect parameters was investigated.

## 2. Materials and Methods

### 2.1. Preparation of TFT Devices

**Figure 1:**
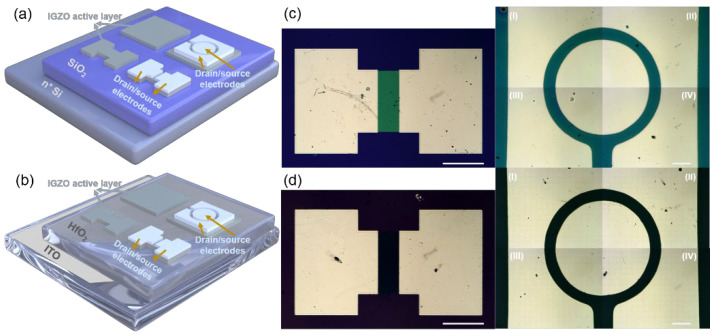
Schematic illustrations of fabrication of an IGZO/SiO_2_/Si thin-film transistor (TFT) (**a**) and an IGZO/HfO_2_/indium tin oxide (ITO) TFT (**b**). Microscope images of the rectangular and circular channel patterns of an IGZO/SiO_2_/Si TFT (**c**) and an IGZO/HfO_2_/ITO TFT. I, II, III, and Ⅳ are four parts of the circular channel pattern (**d**). The scale bar is 200 μm.

In this paper, the commercial ITO conductive glass (HNXC Tech Co., Ltd., Shenzhen, China) with transmittance of 86% and surface resistance of 11 ohm/sq was used as the bottom electrode of TFTs. A hafnium dioxide (HfO_2_) layer as dielectric layer with a thickness of ~170 nm was deposited on the ITO substrate by rf magnetron sputtering for 120 min in argon atmosphere. The growth conditions were set as growth pressure of 8 mTorr, sputtering power of 150 W, and distance between substrate and sputtering target of 100 mm. A Si wafer with a SiO_2_ thickness of 285 nm from HEFEI KEJING Materials Tech Co., Ltd. (Hefei, China) as the bottom gate and insulation layer was used for contrast with the transparent substrates. The colloidal precursors were prepared from indium nitrate (99.99%), gallium nitrate (99.99%), and zinc acetate (99.99%) dissolved in 5 mL of methyl glycol from Shanghai Aladdin Biochemical Technology Co. Ltd. (Shanghai, China) to achieve IGZO solutions with indium, gallium, and zinc molar ratios of 2:1:7. The active layers were prepared by the spin-coating method. The argon plasma was used to remove adsorbed impurities on the substrate surface before a spin-coating process. The purpose was to reduce the thickness of the active layer and form a shorter path for carrier migration. The plasma processing conditions were set as power supply of 75 W and processing time of 10 s. The spin-coating conditions were set as spin speed of 3000 r/min and duration of 30 s. Then, the samples were placed on the hot plate and heated at 90 °C for 3 min to cure the deposited colloid. The active layer was heated to 550 °C at a heating rate of 5 °C/s with air atmosphere in a rapid annealing furnace and kept for 60 min. The thickness of IGZO active layer after annealing treatment was 40 ± 5 nm. A schematic of structure of the IGZO TFTs on the Si/SiO_2_ and ITO/HfO_2_ substrates with rectangular symmetrical and circular drain/source electrodes is shown in Figure 1. The rectangular-patterned active layer and drain/source electrodes were obtained by lithography. The circular electrode structure was obtained by mask evaporation on the prepared IGZO channel layer using an electron beam evaporation system. The rectangular and circular drain/source electrodes were the evaporation layer of aluminum metal with a thickness of ~50 nm. The rectangular channel pattern has a length of ~100 μm and a width of ~300 μm. The channel length of circular pattern TFT devices is also about ~100 μm.

### 2.2. Characterization

The active layers were obtained by a spin-coater (SPS Spin150i, SPS Company, Bienenbuttel, Germany). The microscope images of the rectangular and circular channel patterns were obtained by a Zeiss microscope (AxioScope A1, Carl Zeiss AG). The IGZO active layers were annealed in air atmospheres using a vacuum rapid annealing furnace (RTP-100, UniTemp, Pfaffenhofen, Germany). The dielectric layers were prepared by rf magnetron sputtering (PVD75, Kurt. J. Lesker Company, Jefferson Hills, PA, USA). The rectangular patterned drain/source electrodes and ac-tive layers are graphically etched with a photolithography system (ABM/6/350/NUV/DCCD/M, ABM, Inc., New York, NY, USA). The surface morphology and roughness of active and dielectric layers were characterized by an atomic force microscope (MFP-3D Origin+, Oxford Instruments, Abingdon, UK). The field-effect parameters were measured by a semiconductor parameter measuring instrument (B1500A, Keysight Technologies, Santa Rosa, CA, USA).

## 3. Results and Discussion

**Figure 2:**
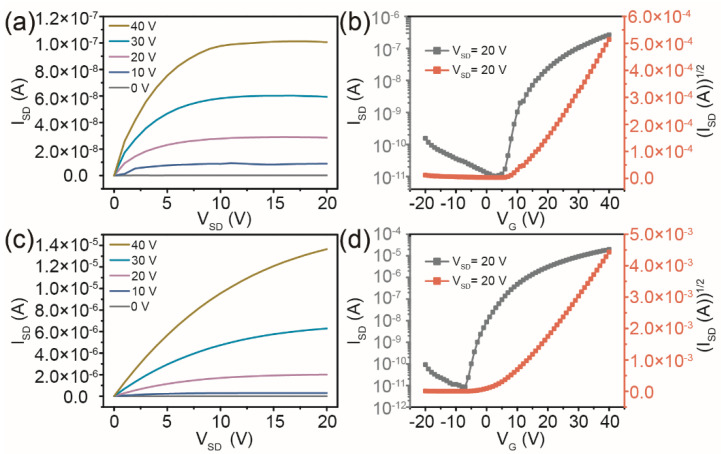
Output characteristics and transfer characteristics of TFTs with rectangular channel patterns (**a**,**b**) and circular channel patterns (**c**,**d**) on the Si/SiO_2_ substrates.

The IGZO channel layer was first deposited on Si/SiO_2_ substrates via a spin-coating process using the IGZO precursor solution. The prepared IGZO film was then annealed at 550 °C, lithographed, and covered with aluminium electrodes to obtain a TFT device with a rectangular channel pattern. The channel length and width of the IGZO/SiO_2_/Si TFT were about 100 μm and 300 μm, respectively. The pristine IGZO channel layer needed to be annealed to remove organic impurities and improve the film quality. The thickness of the prepared IGZO channel layer was about 40 ± 5 nm. The drain current (I_SD_) vs. drain-source voltage (V_SD_) output characteristics of TFTs with rectangular channel patterns at gate voltages (V_G_) from 0 to 40 V is shown in Figure 2a. The curves show the typical n-type TFT performance with the clear transition from linear to saturation behavior. The threshold voltage (V_T_) was estimated by extrapolating the linear portion of the (I_SD_)^1/2^ vs. V_G_ curves at V_SD_ = 20 V in the typical transfer curves of the TFT device with symmetrical rectangular electrodes. The TFT with rectangular pattern channel on a Si/SiO_2_ substrate exhibited an on/off ratio (I_on_/I_off_) of 2.61 × 10^4^ and a V_T_ of 13.5 V. The value of field-effect mobility (μ) can reflect the carrier migration ability of a semiconductor under different electric fields [31]. The μ in the saturation region was evaluated from the following relationship:

(1) $$\mu =\frac{2L}{{\mathrm{WC}}_{i}}{\left(\frac{\partial \sqrt{I_{\mathrm{SD}}}}{\partial V_{G}}\right)}^{2}$$

where L and W are the channel length and width of the IGZO/SiO_2_/Si TFT with a rectangular channel pattern, respectively, and C_i_ is the capacitance per unit area of the SiO_2_ gate insulator with a thickness of about 285 nm. By substituting these parameters into Equation (1), a μ value was obtained for the TFT with a rectangular channel pattern of 0.021 cm^2^/Vs.

**Figure 3:**
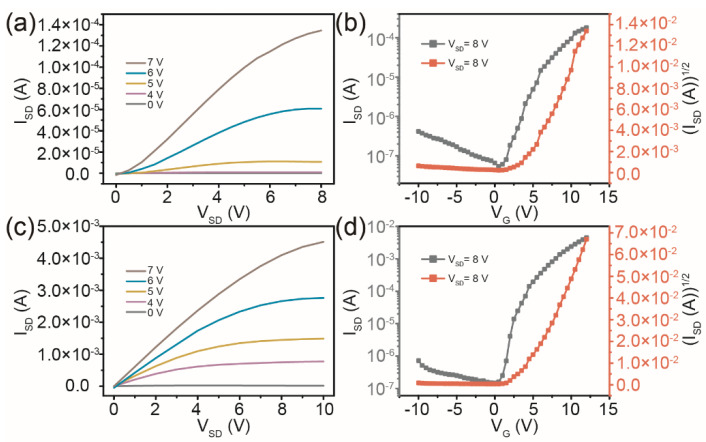
Output characteristics and transfer characteristics of TFTs with rectangular channel patterns (**a**,**b**) and circular channel patterns (**c**,**d**) on ITO/HfO_2_ substrates.

In contrast, the IGZO/SiO_2_/Si TFT device with the circular drain/source electrodes achieves higher I_SD_ in the output characteristic curves, as shown in Figure 2c. The reason is that the carrier injection with circular electrodes in the IGZO channel layers can effectively solve the problem of uneven electric field distribution between the electrode center and edge in traditional symmetrical rectangular structures. The uniform distribution of V_SD_ is more conducive to carrier injection and migration in the channel. The I_on_/I_off_ and the V_T_ estimated by extrapolating the linear portion of the (I_SD_)^1/2^ vs. V_G_ curves at V_SD_ = 20 V in the transfer characteristics of the TFT device with circular channel patterns can be calculated as shown in Figure 3d. It can be seen that the I_on_/I_off_ and V_T_ of the TFT device with circular channel patterns are significantly improved, and the values are 2.04 × 10^6^ and 7.2 V, respectively. For circular drain/source electrodes (as shown in Figure 1c), R_1_ is the radius of the internal source electrode (~500 μm) and R_2_ is the sum of the radius of the internal source electrode and the length of the IGZO channel (~500 + 100 μm) used to calculate the channel width to length ratio (W/L) of TFTs with circular channel patterns, as a function of the W/L change, according to [32]:

(2) $$W/L=\frac{2\pi }{\ln\left(R_{2}/R_{1}\right)}$$

In addition, the value of μ of the IGZO/SiO_2_/Si TFT device with circular electrodes was 0.104 cm^2^/Vs by substituting Equation (2) into Equation (1). The increase in μ can be conducive to improving the switching speed of TFT devices.

With the rapid development of a transparent or translucent display field, the demand for transparent TFTs has gradually increased, and the gate and dielectric layers as the key points of the display driver industry have attracted more attention. In this paper, the ITO transparent conductive films were used as bottom gate electrodes. In addition, the HfO_2_ with high dielectric constant deposited by magnetron sputtering can be selected as the dielectric layer for TFT devices. Similarly, the TFT devices with IGZO channel layers and rectangular and circular drain/source electrodes were prepared on ITO/HfO_2_ substrates, and the output and transfer characteristic curves of the TFTs were characterized as shown in Figure 3. The transition from the linear to the saturation part and the good regulation of the I_SD_ are both obtained in the output characteristic curves of the TFTs with rectangular and circular channel patterns under V_G_ from 0 to 7 V, as shown in Figure 3a,c. The curves show typical n-type transistor performance. However, due to the high conductivity of ITO and the weak compactness of HfO_2_ grown by sputtering at room temperature, a higher I_SD_ and lower applied V_SD_ of the IGZO/HfO_2_/ITO TFTs than those on the traditional Si/SiO_2_ substrates were obtained. The slope value of the (I_SD_)^1/2^ vs. V_G_ curves at V_SD_ = 8 V will be increased for the TFTs with ITO/HfO_2_ substrates, resulting in an increase in estimated μ values. Figure 3b,d show the typical transfer curves I_SD_–V_G_ and (I_SD_)^1/2^–V_G_ at V_SD_ = 8 V of the IGZO/HfO_2_/ITO TFT device with rectangular and circular channel patterns, respectively. The value of μ was derived from a linear fit to the plot of the square root of I_SD_ vs. V_G_. The TFT device with rectangular symmetric drain/source electrodes on ITO/HfO_2_ substrates exhibited a V_T_ of 4.4 V, an I_on_/I_off_ of 3.30 × 10^3^, and a μ of 18.49 cm^2^/Vs. In contrast, a TFT device with the circular channel patterns on the same substrate also showed better field-effect parameters, including a V_T_ of 4.1 V, an I_on_/I_off_ of 2.85 × 10^4^, and a μ of 39.19 cm^2^/Vs. The mobility values reported by different studies are summarized in Table 1. There is a gap between the performance of IGZO TFTs with rectangular and circular channel patterns in this work compared with typical TFTs, but we will improve the electrical properties and stability of the TFT devices and the tolerance of TFTs on ITO substrates to high applied voltage from the perspective of interface modification in the next work.

**Figure 4:**
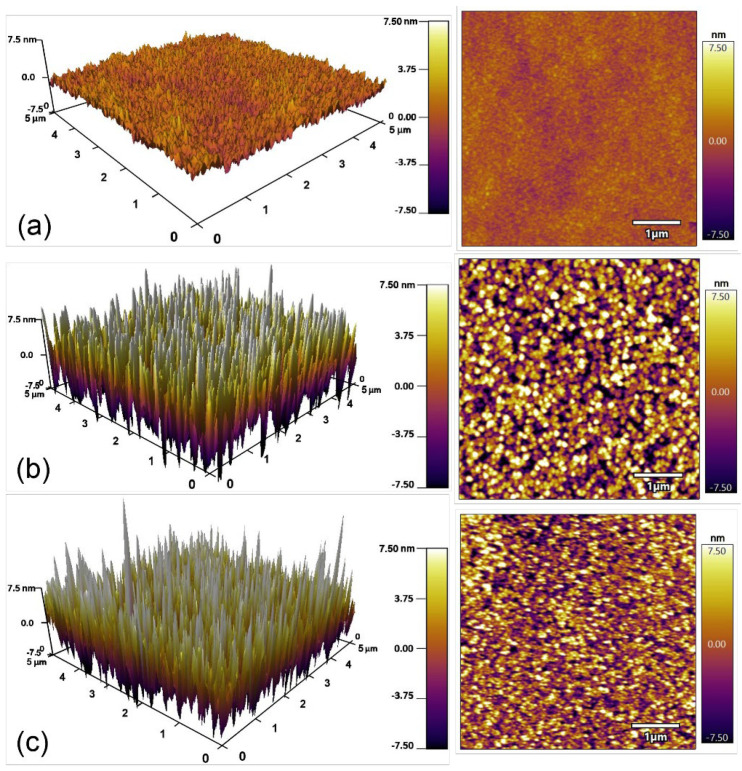
Atomic force microscope images of the IGZO channel layer on the Si/SiO_2_ substrate (**a**), the HfO_2_ dielectric layer deposited on the ITO (**b**), and the IGZO channel layer on the ITO/HfO_2_ substrate (**c**).

In the above, we discussed the variation of electrical performance of IGZO TFT devices on different substrates and with different patterns of drain/source electrodes. For the IGZO channel layer deposited by a spin-coating method, besides the advantage of a simple preparation process, the effect of the spin-coating on surface roughness of the channel layers should also be investigated. In Figure 4, the variation of the IGZO and HfO_2_ surface morphologies on different substrates was analyzed by atomic force microscope. As shown in Figure 4a, the IGZO channel layer by spin-coating on a Si/SiO_2_ substrate has a relatively flat surface with a roughness of 0.796 nm. The very low surface roughness benefited from the preparation of the channel layer from the precursor of the ionic solution. The HfO_2_ layer by magnetron sputtering on the ITO conductive glass has a rougher surface with a roughness of 4.460 nm, as shown in Figure 4b. However, the roughness of the HfO_2_ layer after coating the IGZO channel layer was reduced to 3.797 nm, as shown in Figure 4c. This is because the IGZO precursor solution can be effectively filled into the surface micro-porous structure of the HfO_2_ layer during spin-coating. Reducing the roughness of the surface helps decrease the scattering of carriers at the interface and further improves the transport capacity of carriers in the channel of TFT devices.

## 4. Conclusions

The field-effect performance of the IGZO TFT device with symmetrical rectangular and circular channel patterns on different substrates was investigated in this paper. The electrical properties of TFT devices with circular drain/source electrodes were better than those with a traditional rectangular structure on both substrates, due to the uniform distribution of applied voltages between the electrode center and edge. The uniform distribution of the applied voltage was more conducive to carrier injection and migration in the channel. The TFT with rectangular and circular channel patterns on the Si/SiO_2_ showed a V_T_ value of 13.5 V and 7.2 V, an I_on_/I_off_ value of 2.61 × 10^4^ and 2.04 × 10^6^, and a μ value of 0.021 cm^2^/Vs and 0.104 cm^2^/Vs, respectively. A high dielectric constant HfO_2_ layer on an ITO transparent conductive film was used as the bottom gate and dielectric layer in IGZO TFT devices. The surface roughness of the HfO_2_ layer by sputtering deposition on the ITO can be effectively reduced by spin-coating the IGZO channel layer. This improved interface contact quality will effectively decrease the scattering of carriers at the interface. The TFT with rectangular and circular channel patterns on the ITO/HfO_2_ showed a V_T_ value of 4.4 V and 4.1 V, an I_on_/I_off_ value of 3.30 × 10^3^ and 2.85 × 10^4^, and a μ value of 18.49 cm^2^/Vs and 39.19 cm^2^/Vs, respectively. In the next work, we plan to use transparent metal oxides or metal grids as the drain/source electrodes to solve the problem of full transparency of TFT devices.

## Figures and Tables

**Table 1:** Mobility values of the IGZO TFTs with different substrates and dielectric layers.

Type	Mobility (cm^2^/Vs)	Ref.
Solution-processed IGZO on Si/SiO_2_ substrates	6.41	[33]
Solution-processed IGZO on ITO substrates (all-oxide transparent TFTs)	8	[34]
Solution-processed IGZO with self-assembled nanodielectrics	19.4	[35]
Solution-processed IGZO on Si/SiO_2_ substrates with circular channel patterns	0.104	Our result
Solution-processed IGZO on ITO substrates with circular channel patterns	39.19	Our result

## Data Availability

Not applicable.
